# Robotic Lateral Pelvic Lymph Node Dissection for Advanced Rectal Cancer: Bridging Eastern Surgical Precision and Western Multimodal Strategy

**DOI:** 10.3390/cancers18010077

**Published:** 2025-12-26

**Authors:** Dai Shida

**Affiliations:** Division of Frontier Surgery, The Institute of Medical Science, The University of Tokyo, Tokyo 1088639, Japan; dshida@g.ecc.u-tokyo.ac.jp; Tel.: +81-3-3443-8111; Fax: +81-3-5449-5604

**Keywords:** lateral pelvic lymph node, lateral pelvic lymph node dissection, nerve preservation, rectal cancer, robotic surgery, urinary function

## Abstract

**Background:** Management of lateral pelvic lymph node (LPLN) metastasis in advanced lower rectal cancer has historically exemplified a fundamental East–West divide. In Japan, the Japanese Society for Cancer of the Colon and Rectum (JSCCR) considers LPLN metastasis a regional manifestation requiring lateral pelvic lymph node dissection (LPLND). In contrast, Western practice has long approached LPLN disease as systemic, prioritizing neoadjuvant chemoradiotherapy (nCRT) followed by total mesorectal excision (TME) without additional lateral clearance. **Recent Advances:** Evidence generated from the JCOG0212 trial and subsequent multicenter cohorts has firmly demonstrated that LPLND markedly reduces lateral local recurrence, particularly in patients with radiologically enlarged nodes. These findings have contributed to a paradigm shift: the 2025 European Society for Medical Oncology (ESMO) Guidelines now endorse selective LPLND for suspicious nodes following neoadjuvant therapy, indicating an emerging convergence between Eastern surgical philosophy and Western multimodal treatment strategies. **Surgical Innovation:** Robotic surgery has transformed the technical execution of LPLND. Its stable, high-definition three-dimensional visualization, wristed instruments, and enhanced precision enable meticulous dissection across four anatomically defined planes: the medial plane (uretero-hypogastric fascia), intermediate plane (vesico-hypogastric fascia), lateral plane (pelvic sidewall), and dorsal plane (pelvic floor and lumbosacral trunk/sacral plexus). These features facilitate consistent nerve-sparing surgery, reduce blood loss, and improve postoperative urinary and sexual function compared with conventional laparoscopy or open approaches. Robotic LPLND therefore represents a contemporary synthesis of Eastern surgical precision and Western evidence-based multimodal therapy—offering an integrated pathway toward optimized oncologic control and enhanced functional outcomes.

## Simple Summary

Management of lateral pelvic lymph node (LPLN) metastasis in advanced lower rectal cancer has long differed between Japan and Western countries. In Japan, LPLN metastasis is regarded as a regional extension that can be surgically removed by lateral lymph node dissection (LPLND), whereas Western practice has relied mainly on chemoradiotherapy. Recent clinical evidence, including the JCOG0212 trial, has demonstrated that LPLND can significantly reduce local pelvic recurrence in selected patients. With these findings, Western guidelines have begun to recognize selective LPLND as a valid option, reflecting a growing convergence between Eastern and Western strategies. The introduction of robotic surgery has further enhanced the precision and safety of LPLND, minimizing functional complications while maintaining oncologic radicality. This review summarizes the evolution of global LPLND practice and highlights how robotic technology may bridge East–West differences in the multidisciplinary management of advanced rectal cancer.

## 1. Introduction

Lateral pelvic lymph node (LPLN) metastasis in advanced lower rectal cancer is a critical determinant of local recurrence and overall prognosis. However, its optimal management remains debated across Eastern and Western practices. In Japan, the Japanese Society for Cancer of the Colon and Rectum (JSCCR) has regarded lateral lymph node dissection (LPLND) as an integral component of total mesorectal excision (TME) for clinical T3–T4 lower rectal cancers, aiming to achieve local control beyond the mesorectal plane [1]. In contrast, Western guidelines predominantly favor neoadjuvant chemoradiotherapy (nCRT) followed by TME alone, often dismissing LPLND as unnecessary in the context of multimodal therapy [2,3].

This divergence was re-evaluated following the final analysis of the JCOG0212 trial (published in 2017 [4]), which compared mesorectal excision (ME) alone with ME plus LPLND in stage II/III lower rectal cancer without clinically apparent LPLN metastasis [5,6]. The trial failed to confirm non-inferiority of ME alone, thereby reaffirming the oncologic importance of LPLND for reducing lateral pelvic recurrence. As a result, LPLND has regained clinical attention—especially in patients with radiologically enlarged or persistent LPLNs after nCRT.

Meanwhile, advances in robotic surgery have substantially lowered the technical hurdles associated with LPLND [7,8]. Enhanced instrument articulation, three-dimensional visualization, and tremor reduction enable precise dissection within the deep, narrow pelvis—an area where conventional laparoscopy often struggles [9]. These advantages have broadened the accessibility of LPLND beyond high-volume Japanese centers, stimulating renewed global interest in this procedure [10].

Recent guideline updates further underscore an evolving landscape. The JSCCR 2024 Guidelines present the most refined framework to date, reaffirming indications for LPLND while emphasizing that no validated criteria exist to safely omit the procedure [11]. The update also highlights advances in diagnostic precision, including MRI-based size thresholds and combined dimensional assessment derived from recent multicenter evidence. In addition, new sections on total neoadjuvant therapy (TNT) and non-operative management were introduced; however, both strategies are weakly recommended against, reflecting the limited supporting data in contemporary Japanese practice.

In parallel, Western guidelines have undergone substantive conceptual change. The National Comprehensive Cancer Network (NCCN) [2] continues to conceptualize lateral pelvic disease primarily within a systemic framework, recommending chemoradiation or TNT and reserving LPLND for highly selected patients with persistent or suspicious nodes after multimodal therapy. In contrast, the 2025 European Society for Medical Oncology (ESMO) Guidelines introduce explicit MRI-based criteria for selective LPLND—particularly for patients with persistent internal iliac or obturator nodes despite optimized neoadjuvant treatment [12]. This shift reflects emerging international evidence supporting the oncologic benefit of selective LPLND in appropriately chosen patients.

Collectively, these developments signal a gradual convergence between historically divergent Eastern and Western paradigms. High-resolution MRI, risk-adapted algorithms, and evidence-based selection are reshaping a more unified global approach to lateral pelvic disease in rectal cancer. Against this backdrop, robotic LPLND has emerged as a platform capable of harmonizing technical precision with multimodal oncologic strategy.

The purpose of this review is to synthesize the historical evolution, current evidence, and contemporary guideline updates (JSCCR, NCCN, ESMO), and to delineate the technical principles and distinct advantages of the robotic approach. By integrating Eastern surgical expertise with Western multimodal frameworks, this review aims to clarify the evolving role of robotic LPLND in personalized and globally standardized rectal cancer care.

## 2. Historical Background and Rationale for LPLND

The management of LPLN metastasis in rectal cancer has historically diverged between Japan and Western countries, primarily due to fundamental differences in disease classification and treatment philosophy.

### 2.1. Guideline-Based Concepts of Lateral Lymph Node Dissection in Japan, the United States, and Europe a Decade Ago

The concept of LPLND has historically evolved along distinctly different trajectories in Eastern and Western colorectal oncology. In Japan, LPLN metastasis has long been regarded as a regional extension of rectal cancer rather than a manifestation of systemic disease. This interpretation stems from meticulous anatomic mapping studies conducted in the mid-20th century, which delineated a consistent lymphatic drainage pathway from the lower rectum to the internal iliac and obturator regions. Based on this anatomical rationale, the Japanese surgical community established a philosophy of *en bloc* regional control, culminating in the standardized D3 lymphadenectomy that encompasses the lateral pelvic compartment [13,14].

This surgical philosophy was institutionalized in the JSCCR Guidelines 2016 [15], which recommended routine LPLND for tumors with the lower margin below the peritoneal reflection or with invasion beyond the muscularis propria (cT3–4). An analysis of 2916 cases of rectal cancer in the project study by the JSCCR showed that the LPLN metastasis rate in patients whose lower tumor border was located distal to the peritoneal reflection and whose cancer had penetrated through the rectal wall was 20.1% (only patients who underwent LPLND) [15]. After performing LPLND for the indication mentioned above, the risk of intrapelvic recurrence decreased by 50%, and the 5-year survival rate improved by 8–9% [16]. Thus, the JSCCR classified LPLN metastasis as regional disease (N3) [17], thereby integrating prophylactic or therapeutic LPLND into total mesorectal excision (TME) for locally advanced low rectal cancer.

In contrast, the NCCN 2016 [18] and the ESMO 2013 guidelines [3] conceptualized LPLN involvement as a potential indicator of systemic dissemination. Both Western frameworks prioritized neoadjuvant chemoradiotherapy (nCRT) followed by TME as the standard of care and did not recommend routine LPLND. Instead, LPLND was reserved for highly selected cases—typically when post-nCRT imaging revealed persistent or suspicious lateral nodes. The NCCN emphasized the principles of multimodal therapy and functional preservation, while ESMO underscored the oncologic adequacy of precise mesorectal excision rather than extended lymphadenectomy.

In summary, the JSCCR guidelines established an anatomy-based, region-oriented framework for LPLND, in sharp contrast to the systemic, multimodal philosophy adopted in Western practice during the same period [19].

### 2.2. Evidence from the JCOG0212 Trial (Published in 2017 [4])

The pivotal JCOG0212 randomized controlled trial fundamentally shaped Japan’s modern approach to LPLND for lower rectal cancer. This multicenter phase III trial compared ME alone with ME plus bilateral LPLND in patients with stage II/III lower rectal cancer without clinically apparent LPLN metastasis (defined as a short-axis diameter <10 mm on CT or MRI) [5]. The study failed to confirm the non-inferiority of ME alone to ME plus LPLND in terms of relapse-free survival (RFS), the primary endpoint [4]. Pathological examination revealed LPLN metastasis in 7% of patients despite radiologically negative findings, underscoring the presence of occult lateral spread [5]. Although the 5-year overall survival was comparable between groups (92.6% with LPLND vs. 90.2% without), the addition of LPLND significantly reduced lateral pelvic recurrence (7.4% vs. 12.6%) [4]. These findings confirmed that lateral pelvic disease, even when occult, represents a potentially curable regional entity rather than systemic spread.

ME plus LPLND was associated with longer operative time and greater blood loss, but without a clinically prohibitive increase in severe postoperative complications [5].

Long-term follow-up further supported this conclusion:

The 7-year local relapse-free survival (82.9% vs. 78.9%) and 7-year lateral relapse-free survival (85.3% vs. 80.3%) were both superior in the ME + LPLND group. In a subgroup analysis of clinical stage III disease, relapse-free survival was significantly longer in the LPLND arm [6].

In terms of functional outcomes, autonomic nerve–preserving LPLND did not significantly increase early urinary dysfunction compared with ME alone [20]. However, excessive intraoperative blood loss was identified as a major risk factor for postoperative urinary morbidity [20], emphasizing the need for meticulous hemostatic technique. As for male sexual dysfunction, although overall differences were not statistically significant, moderate-to-severe erectile dysfunction occurred somewhat more frequently in the LPLND group [21].

Collectively, the results of JCOG0212 indicate that routine omission of LPLND cannot be justified in patients with lower rectal cancer even when no enlarged lateral nodes are radiologically evident, given its proven benefit for local control. Accordingly, JCOG0212 established LPLND as the standard of care in Japan for clinical stage II/III low rectal cancer in the absence of preoperative chemoradiotherapy (nCRT) [22].

However, its applicability remains limited for patient populations excluded from the trial, such as those with bulky tumors, deep extramural invasion, or threatened circumferential margins, in whom LPLND alone without nCRT may be suboptimal [22]. Furthermore, the clinical value of LPLND in LPLN-negative cases after preoperative chemoradiotherapy remains uncertain, and the additional invasiveness of combined local control strategies must be carefully weighed against potential benefits.

Collectively, JCOG0212 established lateral pelvic disease as a potentially curable regional entity, providing the first level I evidence supporting LPLND for local control.

### 2.3. Japanese Paradigm: Evolution of the JSCCR Guidelines on Lateral Pelvic Lymph Node Dissection (2009–2024)

The JSCCR has consistently regarded LPLN metastasis as a regional rather than systemic disease [23]. This fundamental concept has guided Japan’s surgical philosophy for decades. The JSCCR guidelines from 2009 to 2024 chronicle a gradual transition from empiricism to evidence-based precision, anchored by the results of the JCOG0212 trial.

#### 2.3.1. JSCCR Guidelines 2009–2016: Foundation in Retrospective Evidence

The early JSCCR guidelines endorsed LPLND for tumors below the peritoneal reflection with invasion beyond the muscularis propria (T3 or deeper). Retrospective multicenter studies in Japan have reported that 15–20% of patients with lower rectal cancer harbor LPLN metastases [16,24,25]. Although Western oncologic paradigms have traditionally regarded LPLN involvement as a marker of systemic disease associated with poor prognosis, several Japanese studies demonstrated that R0 resection—including LPLND—can achieve 5-year overall survival rates of 45–55% in selected patients [16,24,25,26], particularly those with a limited number of metastatic nodes [27] or metastases confined to the internal iliac region [23].

A nationwide propensity score-matched analysis by the JSCCR, including patients with pT3/T4 lower rectal cancer from 1995 to 2004, revealed superior 5-year overall survival in the LPLND group compared with the non-LPLND group (68.9% vs. 62.0%) [28]. In addition, a multicenter study showed that patients with mildly enlarged LPLNs on preoperative imaging derived a survival benefit from LPLND [29]. Compared with D3 dissection of the inferior mesenteric root nodes, the therapeutic impact of LPLND was found to be greater in selected cases [26,30].

Taken together, despite the inherent limitations of retrospective data, these consistent findings established TME plus LPLND as the national standard of care in Japan during this period, based on its contribution to improved pelvic control and survival in selected patients.

#### 2.3.2. JSCCR Guidelines 2019: Integration of JCOG0212—Evidence-Based Validation

The 2019 revision of the JSCCR Guidelines represented a pivotal turning point, as it formally incorporated the evidence generated by the landmark JCOG0212 trial. Based on these results, the recommendations for LPLND were stratified according to the clinical status of lateral nodes:

- Clinically positive LPLN: Strong recommendation
- Clinically negative LPLN (prophylactic): Weak recommendation

These evidence-based revisions acknowledged that, although the survival benefit of LPLND remains limited, its addition to TME significantly reduces the risk of local pelvic recurrence. Consequently, LPLND was retained as a standard surgical option in Japan for locally advanced low rectal cancer without preoperative chemoradiotherapy, reflecting a balanced interpretation of efficacy and invasiveness grounded in level I evidence.

In summary, the 2019 JSCCR revision marked the transition from empiricism to level I evidence, validating LPLND as a means of improving local control while acknowledging its limited impact on overall survival.

#### 2.3.3. JSCCR Guidelines 2024: Toward Precision and Selectivity [11]

Following two decades of incremental refinement, the 2024 JSCCR Guidelines introduced the most sophisticated framework to date, emphasizing precision, selectivity, and evidence-based adaptation of LPLND in rectal cancer surgery. The 2024 JSCCR guidelines reaffirmed the two key recommendation for LPLND. First, LPLND is recommended when preoperative or intraoperative findings indicate the presence of lateral pelvic lymph node metastasis (Recommendation 1, Evidence Level C). Second, even in the absence of detectable metastasis, LPLND is recommended as it may help reduce local recurrence, although its survival benefit remains limited (Recommendation 2, Evidence Level B).

In the 2024 revision [11], the commentary section was updated to clarify that the criteria for safely omitting LPLND have not yet been established. The description of ongoing multicenter studies conducted by the JSCCR was also revised to incorporate the most recent findings. Although a randomized controlled trial conducted in Japan reported that preoperative radiotherapy could achieve a therapeutic effect comparable to LPLND [31], its small sample size and lack of external validation limit the generalizability of the results. Furthermore, Accurate preoperative diagnosis of LPLN metastasis remains a major challenge. A retrospective JSCCR study demonstrated that using a 5 mm cutoff for the short-axis diameter on magnetic resonance imaging (MRI) yielded better diagnostic performance than the conventional 10 mm threshold [32], though this also underscored the limitations of size-based criteria using standard MRI protocols. To address this, a multicenter prospective study led by the JSCCR is currently underway to establish diagnostic criteria based on high-resolution MRI. Preliminary findings indicate that combining both long- and short-axis measurements enables detection of metastatic nodes with >90% sensitivity, representing a significant step toward precision imaging and individualized surgical decision-making [33].

The 2024 JSCCR Guidelines also newly introduced sections on total neoadjuvant therapy (TNT) and non-operative management (NOM) for rectal cancer, both of which are weakly not recommended at this stage [11]. These additions reflect an increasing awareness of evolving global treatment paradigms and the need to integrate them carefully into Japanese clinical practice.

Overall, the 2024 update highlights Japan’s shift toward risk-adapted, imaging-guided, and multidisciplinary use of LPLND, balancing oncologic benefit with procedural invasiveness.

### 2.4. United States’ Paradigm: Evolution of NCCN Guidelines on Lateral Lymph Node Dissection (2018–2025)

In the United States, LPLN involvement has traditionally been viewed through the lens of systemic disease, with management centered on multimodal therapy rather than extended surgical resection. The NCCN guidelines, which have profoundly shaped Western oncologic strategy, consistently prioritize neoadjuvant chemoradiotherapy (CRT) or total neoadjuvant therapy (TNT) followed by high-quality total mesorectal excision (TME). From 2018 through 2025, the NCCN has maintained the position that routine LPLND is not recommended, reflecting the belief that LPLN metastasis represents systemic dissemination rather than a locoregional process amenable to surgical clearance.

Earlier NCCN versions (through 2019) discouraged extended nodal dissection beyond the standard TME plane—particularly into the obturator or internal iliac regions—because of concerns regarding longer operative time, greater blood loss, and higher rates of urinary and sexual dysfunction. Instead, locoregional control of the lateral compartment was largely entrusted to radiotherapy. The guidelines have consistently recommended including the internal iliac nodes within the target volume for standard preoperative CRT, and, for anteriorly invasive T4 tumors, extending coverage to the external iliac nodes (2022–2025). This approach underscores the NCCN’s preference for radiologic rather than surgical management of the lateral pelvis.

Beginning in 2020, the NCCN Guidelines introduced an important refinement, explicitly addressing surgical management for patients with predicted positive margins or suspected LPLN involvement. The guidelines stated that “*the treating surgeon should be experienced in rectal cancer surgery, and specifically with TME. For patients with predicted positive margins based on preoperative imaging, or lateral pelvic lymph node involvement, the surgeon should be experienced in extended resections beyond the TME plane and have a multidisciplinary team available if necessary*.” [34]. This marked the first formal acknowledgment within the NCCN framework that selected patients with LPLN involvement may require extended resection beyond the standard TME field. The statement underscored that such procedures should only be performed by surgeons with expertise in complex pelvic surgery and access to multidisciplinary support, emphasizing the importance of institutional capability and surgical judgment in managing disease spread within the lateral compartment. Notably, this principle has been retained unchanged through the most recent 2025 edition of the guidelines [2].

Further evolution was observed in subsequent updates (2023–2025), which incorporated the principles of total neoadjuvant therapy (TNT) and introduced the concept of non-operative management (NOM) or “watch-and-wait” for patients achieving a clinical complete response [2]. Although these sections pertain to organ preservation rather than lateral dissection, they reflect a broader NCCN shift toward individualized treatment balancing oncologic control with functional outcomes.

In summary, the NCCN guidelines over this decade reflects a gradual shift from uniform avoidance of lateral dissection toward conditional selectivity informed by radiologic findings, surgical expertise, and multidisciplinary collaboration. What was once a categorical rejection of LPLND has matured into a more individualized strategy—one that bridges Western multimodal principles with Eastern evidence for precision surgery.

### 2.5. Europe’s Paradigm: Evolution of ESMO Guidelines on Lateral Lymph Node Dissection (2017–2025)

The European Society for Medical Oncology (ESMO) guidelines have undergone a remarkable conceptual evolution over the past decade—from non-recommendation in 2012 to selective endorsement in 2025—reflecting a growing convergence between Western and Eastern philosophies in the management of LPLN disease.

In the 2017 version [35], ESMO characterized LPLND as “*rarely practiced in Europe*”, advising consideration only for patients with persistently enlarged lateral nodes following neoadjuvant chemoradiotherapy (CRT). The prevailing Western strategy emphasized preoperative radiotherapy, either long-course CRT or short-course RT, as the principal method to sterilize microscopic disease and minimize surgical morbidity. This position implicitly contrasted with the Japanese approach, which regarded enlarged LPLNs as potentially resectable regional metastases rather than systemic spread.

The 2025 ESMO update represents a distinct paradigm shift driven by emerging evidence [12]. The guidelines stated that “*in case of suspected lymph node metastases, lateral pelvic nodes (internal iliac and obturator lymph nodes) considered to be locoregional disease may also require resection*”. Thus, the new guidelines explicitly recommend selective LPLND for patients with radiologically suspicious nodes meeting defined size thresholds—specifically, those with a short-axis diameter ≥7 mm before neoadjuvant therapy. These criteria are supported by Level IV evidence and carry a Grade A recommendation. The update draws heavily on data from the Lateral Node Study Consortium, which demonstrated that in patients with LPLNs ≥7 mm, local recurrence occurred in 19.5% after CRT plus TME alone, compared with only 5.7% when LPLND was added [36]. Restaging MRI is now emphasized as a critical decision tool: if nodes shrink from ≥7 mm to ≤4 mm after CRT, LPLND may be omitted, whereas persistent internal iliac nodes >4 mm warrant surgical clearance to optimize local control [37].

In summary, the 2025 ESMO guidelines signify a major inflection point in European rectal cancer management. While earlier versions relied exclusively on CRT or total neoadjuvant therapy for lateral control, the current framework acknowledges that multimodal therapy alone may fail to eradicate disease in enlarged or persistent lateral nodes. This shift underscores a data-driven, selective adoption of surgical principles long established in Eastern practice, paving the way for greater international harmonization in the treatment of advanced rectal cancer.

### 2.6. Comparison of LPLND Recommendations Across NCCN (2025), JSCCR (2024), and ESMO (2025) Guidelines

Table 1 summarizes the current recommendations on LPLND across the JSCCR (2024), NCCN (2025), and ESMO (2025) guidelines, highlighting the ongoing convergence between Eastern and Western paradigms in rectal cancer management. Collectively, these guidelines demonstrate a gradual alignment toward precision, risk-adapted surgery for patients with lateral node-positive rectal cancer.

The JSCCR (2024) continues to define LPLN metastasis as a regional disease entity, recommending LPLND—either prophylactic or therapeutic—for lower rectal tumors located below the peritoneal reflection. In contrast, the NCCN (2025) retains a more conservative stance, discouraging routine LPLND but allowing selective dissection or biopsy in cases with clinically apparent or radiologically persistent nodal disease after optimized chemoradiotherapy (CRT) or total neoadjuvant therapy (TNT). The ESMO (2025) guidelines now occupy an intermediate position, explicitly endorsing selective LPLND for high-risk patients based on MRI-determined size criteria—thereby bridging the historical gap between the surgical emphasis of the East and the radiation-based strategies of the West.

A unifying element across all three guidelines is the central role of high-resolution pelvic MRI for pre- and post-treatment evaluation, which enables individualized, evidence-based decision-making regarding LPLND. The comparative analysis of JSCCR 2024, NCCN 2025, and ESMO 2025 underscores a clear trend toward risk-stratified, multidisciplinary management of the lateral compartment.

What was once a pronounced East–West divide has evolved into a shared, data-driven algorithm that emphasizes selective LPLND guided by advanced imaging and refined through minimally invasive—particularly robotic—techniques. This convergence reflects a broader paradigm shift in modern rectal cancer care: the fusion of surgical innovation with oncologic precision to achieve both radical disease control and preservation of postoperative function.

## 3. The Rise of Robotic LPLND: Bridging Eastern Surgical Precision and Western Multimodal Strategy

### 3.1. Evolution of Minimally Invasive LPLND

The adoption of minimally invasive techniques has transformed rectal cancer surgery; however, LPLND remained technically formidable for many years. Early reports of laparoscopic LPLND from Japan and Korea in the early 2000s demonstrated feasibility but highlighted inherent limitations of conventional laparoscopy—restricted instrument maneuverability, unstable camera control, limited visualization, and ergonomic strain within the deep and narrow pelvis. These constraints contributed to prolonged operative times, greater blood loss, and a steep learning curve, slowing global dissemination even among experienced colorectal surgeons. Concerns regarding autonomic nerve injury and postoperative urinary or sexual dysfunction further compounded hesitancy.

The introduction of robotic platforms fundamentally altered this landscape. Robotic systems offer three-dimensional high-definition visualization, tremor filtration, motion scaling, and multi-jointed instruments capable of articulating with human wrist-like dexterity. These capabilities permit precise dissection around the internal iliac vessels, obturator nerve, pelvic splanchnic nerves, and other critical neurovascular structures in the lateral compartment. As a result, robotic surgery enables consistent nerve-sparing LPLND with enhanced control and safety, addressing many of the limitations that historically challenged the laparoscopic approach.

High-volume centers across Asia—particularly in Japan, Korea, and China—have reported the feasibility and safety of robotic-LPLND. Compared with conventional laparoscopy, Robotic LPLND has been associated with reduced intraoperative blood loss, comparable or shorter hospital stays, and improved postoperative urinary function [38,39,40,41,42,43,44]. Enhanced optical clarity also facilitates identification of key fascial landmarks, including the vesico-hypogastric and parietal pelvic fascia, enabling dissection along precise oncologic planes while minimizing nerve injury. Collectively, these advances have significantly reduced the functional morbidity historically associated with LPLND and have paved the way for its broader international adoption beyond specialized Eastern institutions.

### 3.2. Technical and Clinical Advantages of Robotic LPLND

Despite the generally longer operative time, robotic LPLND confers several noteworthy technical and short-term clinical advantages compared with the laparoscopic approach. One of the most consistent findings is the enhanced quality of nodal dissection. Multiple studies have demonstrated that robotic LPLND results in a greater number of harvested lateral pelvic nodes [38,42,45,46], the benefit most evident in anatomically demanding regions such as the distal internal iliac (station 263D) and obturator basins (station 283) [43,46], where the robotic platform facilitates more comprehensive nodal clearance than laparoscopy. Another important advantage relates to postoperative genitourinary function. The precision afforded by the robotic system contributes to more reliable preservation of the autonomic nerves, and patients undergoing robotic LPLND tend to experience faster recovery of urinary function. Several reports have noted a significantly lower incidence of postoperative urinary retention requiring catheter reinsertion compared with laparoscopic LPLND [9,46], a finding that has been corroborated by recent meta-analytic evidence [38]. The robotic approach also appears to improve intraoperative safety, particularly with respect to hemostasis. Estimated blood loss is consistently lower in robotic series, reflecting superior control of small-caliber vessels encountered during deep pelvic dissection [46]. From a technical standpoint, the advantages of robotic surgery—wristed instrument articulation, motion scaling, high-definition three-dimensional visualization, and a stable camera platform—translate into more effective and deliberate maneuvers within the narrow pelvic cavity. These features facilitate meticulous dissection around intricate neurovascular structures, enabling precise lymphatic tissue removal while minimizing traction or thermal injury to the pelvic autonomic plexus [43,46]. Such benefits are especially relevant in male patients or those with a narrow bony pelvis, in whom exposure and instrument maneuverability are inherently limited during standard laparoscopy.

Although robotic surgery is associated with higher upfront procedural costs, current evidence demonstrates oncologic outcomes comparable to laparoscopic approach, with modest improvements in disease-free survival and locoregional control in selected patients, but no consistent advantage in overall survival [47]. Importantly, several studies suggest that these higher initial costs may be partially offset by reduced conversion rates, lower postoperative morbidity, and shorter hospital stays, particularly in complex pelvic procedures such as LPLND [48].

### 3.3. Integrating Robotic Precision into Global Rectal Cancer Strategy

Robotic LPLND has emerged as a pivotal modality in harmonizing the historically divergent Eastern and Western treatment paradigms for advanced lower rectal cancer. In Japan and other Asian countries, the technology has accelerated the transition from open to minimally invasive and function-preserving LPLND while maintaining oncologic thoroughness. Conversely, in Western practice, where management has been anchored in chemoradiation and selective surgical intervention, robotic LPLND is increasingly adopted for patients with radiologically persistent or enlarged lateral nodes after neoadjuvant treatment. This shift parallels updates in major guidelines, including the 2025 ESMO criteria that endorse selective LPLND in appropriately staged disease [12].

Although current evidence remains predominantly retrospective and randomized comparisons with laparoscopic LPLND are still absent, available data consistently show reduced morbidity, improved postoperative genitourinary function, and comparable oncologic outcomes with robotic assistance. These benefits, combined with enhanced precision in deep pelvic dissection, underscore the feasibility of incorporating robotic LPLND into contemporary treatment algorithms across diverse healthcare systems. In this evolving global landscape, robot LPLND represents a practical synthesis of Eastern surgical precision and Western multidisciplinary oncologic strategy, offering a shared technological platform for standardized yet individualized management of advanced rectal cancer.

As the worldwide adoption of robotic platforms continues to expand, the next essential step is a detailed understanding of the pelvic anatomy and procedural standardization required to perform safe and effective robotic LPLND—topics addressed in the following section.

## 4. Robotic Lateral Pelvic Lymph Node Dissection

### 4.1. Essential Anatomy for Robotic Lateral Pelvic Lymph Node Dissection

**Figure 1:**
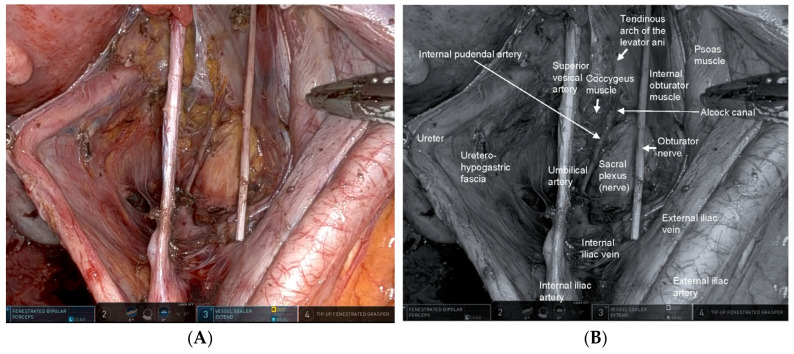
Right-sided lateral pelvic anatomy after robotic lateral pelvic lymph node dissection (LPLND). (**A**) Intraoperative robotic view demonstrating the post-dissection anatomy of the right lateral pelvic compartment with clear exposure of the obturator space. The right internal pudendal artery is visualized continuously from its course within Alcock’s canal to its exit from the pelvis, serving as a key landmark for safe dissection along the distal internal iliac system. (**B**) Corresponding monochrome image annotated with essential right-sided anatomic landmarks—including the obturator nerve, internal iliac vessels, and internal pudendal artery—highlighting the critical surgical planes encountered during LPLND.

**Figure 2:**
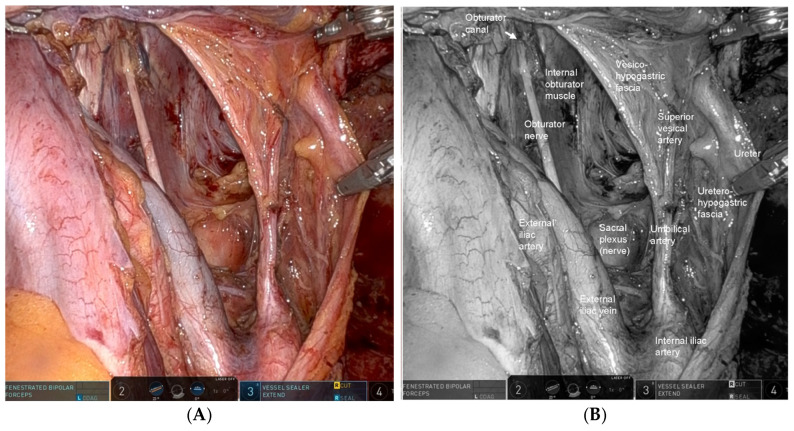
Left-sided lateral pelvic anatomy after robotic lateral pelvic lymph node dissection (LPLND). (**A**) Intraoperative robotic view demonstrating the left lateral pelvic compartment after complete nodal clearance, with clear visualization of the vesico-hypogastric fascia—a major landmark defining the medial boundary of dissection—and the uretero-hypogastric fascia, which guides preservation of pelvic autonomic nerves. (**B**) Corresponding monochrome image labeled with key left-sided anatomic structures, including the vesico-hypogastric fascia, the boundaries of the obturator and internal iliac nodal stations, illustrating the precise dissection zones relevant to oncologic LPLND.

The key to robotic LPLND lies in accurate recognition of the fascial planes, major vessels, and critical nerves that define the internal iliac (station 263) and obturator (station 283) lymph node basins [17]. Contemporary understanding of the pelvic fascial architecture provides a clear framework for safe and systematic dissection [49]. Robotic LPLND is structured around four reproducible planes—the medial (uretero-hypogastric fascia), intermediate (vesico-hypogastric fascia), lateral (pelvic wall), and dorsal (pelvic floor and lumbosacral trunk/sacral plexus)—which together map the three-dimensional boundaries of the lateral pelvic compartment [50]. Mastery of these layers allows surgeons to perform oncologically complete node clearance while preserving autonomic and somatic nerve integrity. Figure 1 and Figure 2 depict the right- and left-sided pelvic anatomy after robotic LPLND, illustrating the layered spatial relationships that guide this approach.

#### 4.1.1. Medial Plane—The Uretero-Hypogastric Fascia [50,51,52,53]

The uretero-hypogastric fascia forms the medial boundary of the LPLND field and constitutes one of the most important anatomical planes for functional preservation during pelvic autonomic nerve–sparing surgery [51]. Anatomically, the uretero-hypogastric fascia is a thin but distinct membranous structure that envelops the ureter, the hypogastric nerve, the pelvic splanchnic nerves, and the pelvic autonomic nerve plexus (Figure 1). By encapsulating these structures within a unified neurovascular sheath on the medial side of the pelvic sidewall, the uretero-hypogastric fascia provides a reproducible and protective interface separating the visceral autonomic pathway from the lateral lymphatic basin. In terms of continuity, the uretero-hypogastric fascia is contiguous superiorly with the ureteral fascia derived from the anterior renal fascia, and inferiorly it merges with the pre-hypogastric nerve fascia within the TME plane [52]. This cranio-caudal continuity allows surgeons to maintain a consistent medial fascial plane during combined TME and LPLND, thereby preventing inadvertent entry into the autonomic nerve compartment. From a surgical standpoint, meticulous dissection along the outer surface of the uretero-hypogastric fascia allows the ureter and the autonomic nerves to be mobilized together as a single fascial sheet and retracted medially. This maneuver safely isolates these structures from the nodal basin of the internal iliac region (station 263), minimizing the risk of nerve injury and postoperative dysfunction. Regarding vascular anatomy, the middle rectal artery often traverses the uretero-hypogastric fascia to supply the rectum, which may restrict the dissection layer. Dividing this artery facilitates a wider exposure of the pelvic nerve plexus and allows safe continuation of the dissection plane. Separating the potential space between the uretero-hypogastric fascia and the vesico-hypogastric fascia (described below) allows for systematic dissection of the internal iliac nodes (station 263).

#### 4.1.2. Intermediate Plane—The Vesico-Hypogastric Fascia [50,51]

The vesico-hypogastric fascia is a prominent anatomical landmark that plays a pivotal role in robotic LPLND, serving as the intermediate fascial plane separating the internal iliac and obturator lymph node regions. It provides a crucial reference for orienting the dissection and maintaining surgical precision in the deep pelvic cavity. Anatomically, the vesico-hypogastric fascia is a dense, well-defined fascial membrane that envelopes the visceral branches of the internal iliac artery and their corresponding venous tributaries, namely, the umbilical artery, superior vesical artery, and inferior vesical vessels, which collectively supply the urinary bladder and pelvic organs. Extending as a broad fascial “curtain” between the internal iliac system and the lateral bladder wall, it compartmentalizes the pelvic vasculature and contributes to the fascial framework supporting the pelvic viscera (Figure 2). From a surgical perspective, the vesico-hypogastric fascia delineates the medial boundary of the obturator region (station 283) [17] and serves as an indispensable guide for lymph node dissection. By maintaining dissection along the avascular plane lateral to the vesico-hypogastric fascia and medial to the obturator lymphatic package, the surgeon can safely peel the obturator fat tissue away from the preserved visceral vessels, minimizing the risk of vascular or neural injury. In gynecological terminology, the region corresponding to the vesico-hypogastric fascia is partially synonymous with the cardinal ligament, reflecting the shared fascial architecture supporting the pelvic viscera [49]. This cross-disciplinary anatomical correlation reinforces the concept of the vesico-hypogastric fascia as a key structural element that integrates the vascular, fascial, and lymphatic systems of the pelvis.

#### 4.1.3. Lateral Plane—The Pelvic Wall

The lateral plane of the obturator region is defined by the muscular surface of the pelvic wall, forming the lateral boundary opposite the vesico-hypogastric fascia. This plane is primarily composed of the internal obturator muscle, with contributions from the psoas muscle in its more cranial portion. Developing this plane is essential for establishing the full lateral extent of the obturator lymph node compartment and for achieving an anatomically precise lymphadenectomy. Dissection along the internal obturator muscle creates a broad and stable working surface, enabling clear visualization of the pelvic wall landmarks. Within this region course the obturator nerve and its accompanying vessels, the obturator artery and vein, which traverse the obturator canal (Figure 2) toward the medial thigh. During robotic LPLND, the obturator nerve, typically situated between the external and internal iliac venous systems, is carefully identified and skeletonized from surrounding lymphoadipose tissue. Preservation of the obturator nerve is essential for preventing postoperative impairment of thigh adduction and sensory loss along the medial aspect of the thigh, thereby maintaining functional mobility and patient safety.

In contrast, the obturator artery and vein are generally divided near their entrance into the obturator foramen to ensure complete removal of nodal tissue within the compartment. Precise identification of these vessels allows the surgeon to balance oncologic completeness with safe hemostasis, particularly in the deep and narrow confines of the lateral pelvic space.

Accurate recognition of the pelvic wall’s muscular, neural, and vascular structures enables safe, nerve-preserving, and oncologically adequate clearance along this lateral plane. Robotic high-definition magnification and stable instrument control further enhance the surgeon’s ability to perform meticulous dissection in this constrained anatomic corridor, reinforcing both procedural safety and oncologic rigor.

#### 4.1.4. Dorsal Plane—The Pelvic Floor and the Lumbosacral Trunk/Sacral Plexus

At the dorsal and caudal aspects of the LPLND, the deepest boundary of the lateral compartment is defined by neuro-muscular structures located along the pelvic sidewall. The lumbosacral trunk (L4–L5), which descends into the pelvis to join the proximal sacral plexus (S1–S3) and eventually contribute to the sciatic nerve, becomes visible as a thick, whitish nerve bundle covered by a thin fascial membrane. Preservation of this membranous layer is essential, as it protects the lumbosacral trunk (L4–L5)—a major contributor to the sciatic nerve—from traction or thermal injury. Damage to these fibers can result in postoperative neuropathy characterized by weakness of ankle dorsiflexion and plantarflexion, sensory disturbances along the lower leg and foot, and broader deficits associated with sciatic nerve dysfunction.

Inferiorly, the coccygeus and levator ani muscles constitute the pelvic floor and demarcate the caudal limit of the dissection. At the deepest caudal region, the internal pudendal artery, a distal branch of the internal iliac artery, courses dorsal to the coccygeus muscle and continues toward the Alcock canal. Identification of the internal pudendal artery provides a reliable indicator that the caudal dissection has reached its anatomical endpoint and helps confirm the completeness of the deep lateral compartment clearance.

Within the internal iliac region (station 263), the superior vesical artery, which arises from the patent proximal segment of the umbilical artery, serves as a reliable anatomical boundary separating the proximal and distal internal iliac nodal subgroups, as described in the Japanese Classification of Colorectal, Appendiceal, and Anal Carcinoma [17]. The proximal nodes (station 263P) are located between the bifurcation of the internal iliac artery and the origin of the superior vesical artery, whereas the distal nodes (station 263D) lie below the level of the superior vesical artery. The distal internal iliac region (station 263D) is regarded as the most frequent site of metastasis within the lateral compartment [54].

Complete clearance of the distal internal iliac nodes requires precise, nerve-sparing dissection along the uretero-hypogastric fascia, which provides the fascial interface between the lymphatic–fatty tissue and the inferior vesical vessels together with their accompanying autonomic nerve fibers near the pelvic floor. This step constitutes the deepest and most technically demanding portion of LPLND. The magnified three-dimensional visualization and instrument stability afforded by robotic surgery are especially advantageous in this region, enabling preservation of pelvic autonomic pathways while ensuring comprehensive oncologic removal of the distal internal iliac nodal basin.

### 4.2. Step-by-Step Procedure of Robotic LPLND

The complexity of robot LPLND arises from the need to accurately define surgical planes that allow for radical lymphadenectomy—specifically targeting internal iliac nodes (station 263) and obturator nodes (station 283)—while preserving the autonomic nervous system and major pelvic vessels. Robotic technology, with its 3D magnified vision and wristed instruments, enables the precise identification and dissection of these fine anatomical structures. The standardized procedure is performed along four key anatomical planes as previously described. The following section provides a step-by-step technical overview, emphasizing critical maneuvers in high-risk areas and strategies for functional preservation.

- Step 1: Establishing the Medial Boundary (autonomic nervous system preservation)

The first phase focuses on identifying and mobilizing the autonomic nerves enclosed within the uretero-hypogastric fascia. The ureter is first identified above the bifurcation of the common iliac artery and mobilized caudally. The uretero-hypogastric fascia containing the ureter and the autonomic nerves is then mobilized and retracted medially [49]. The dissection field may be limited by the middle rectal artery, which often penetrates the uretero-hypogastric fascia toward the rectum. Recognizing and dividing this vessel allows wider exposure of the pelvic nerve plexus. This mobilization should extend caudally beyond the S4 level to avoid nerve injury and ensure complete clearance of distal internal iliac nodes (station 263D).

b. Step 2: Obturator nodes Dissection (station 283)

Station 283 nodes are located between the pelvic wall (the lateral plane) which is formed primarily by the internal obturator muscle and the outer surface of the vesico-hypogastric fascia (the outer side of the intermediate plane). The lateral boundary is established by exposing the external iliac artery and vein and extending the avascular plane dorsally toward the internal obturator muscle, creating a wide dorsal–caudal working space. During this step, the obturator nerve and the obturator artery and vein, which arise as parietal branches of the internal iliac vessels, are encountered. The obturator nerve (L2–L4) traverses the compartment and exits through the obturator foramen and must be preserved throughout the dissection. In contrast, the obturator artery and vein are typically divided distally near the obturator canal to secure adequate nodal clearance. The medial boundary is an avascular plane between the vesico-hypogastric fascia and the obturator fat. Medial retraction of the umbilical artery places tension on the vesico-hypogastric fascia, facilitating safe, controlled separation of this plane and improving exposure of the pelvic floor. Once both boundaries are defined, the nodal tissue between the pelvic wall and the vesico-hypogastric fascia is dissected *en bloc*. The course of the obturator nerve is confirmed proximally near the confluence of the internal and external iliac vessels, and meticulous technique—including minimizing thermal spread—is essential to avoid nerve injury.

Although the order of Step 2 (obturator dissection) and Step 3/4 (internal iliac dissection) may be interchanged, our approach begins with the obturator compartment. Defining both the inner and outer boundaries of the nodal field early in the procedure provides better spatial orientation and facilitates prompt hemostasis in case of unexpected bleeding, thus contributing to a safer and more controlled LPLND.

c. Step 3: Proximal Internal Iliac Nodes Dissection (station 263P)

This step addresses the proximal internal iliac nodes (station 263P), situated between the preserved uretero-hypogastric fascia (medial plane) and the vesico-hypogastric fascia (intermediate plane). After widely developing this space to expose the proximal compartment, dissection proceeds along the internal iliac artery and vein above the superior vesical artery, which demarcates the boundary between the proximal and distal nodal basins [17]. From the bifurcation of the common iliac vessels to the origin of the superior vesical artery, lymphatic–fatty tissue is retracted ventrally as the anterior and medial aspects of the internal iliac vessels are sequentially unveiled, allowing systematic and anatomically guided clearance of the station 263P nodes.

d. Step 4: Distal Internal Iliac Nodes Dissection (263D)—Balancing Oncologic Radicality and Functional Preservation

Distal internal iliac nodes (station 263D), located below the superior vesical artery and surrounding the inferior vesical vessels, represent the most frequent site of lateral pelvic node metastasis and therefore require meticulous attention [49,50,51,54]. When metastatic involvement is not suspected, the inferior vesical vessels and adjacent autonomic nerves are preserved, and lymphatic–fatty tissue is sharply separated from the vessel walls to minimize the risk of postoperative urinary dysfunction [55]. In contrast, when metastatic nodes are inseparable from or invading the inferior vesical vessels, *en bloc* resection of the involved vascular segment may be necessary to achieve adequate oncologic clearance; even in such cases, preserving at least one major vesical branch, when anatomically feasible, is recommended. If vessel sacrifice cannot be avoided, careful separation of the autonomic nerves from the vascular root may allow preservation of neural continuity, although direct tumor invasion may necessitate limited resection of the uretero-hypogastric fascia, with the understanding that autonomic nerve sacrifice significantly increases the risk of urinary sequelae [55].

**Figure 3:**
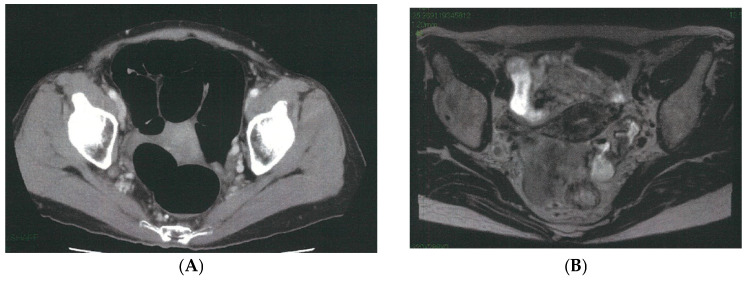

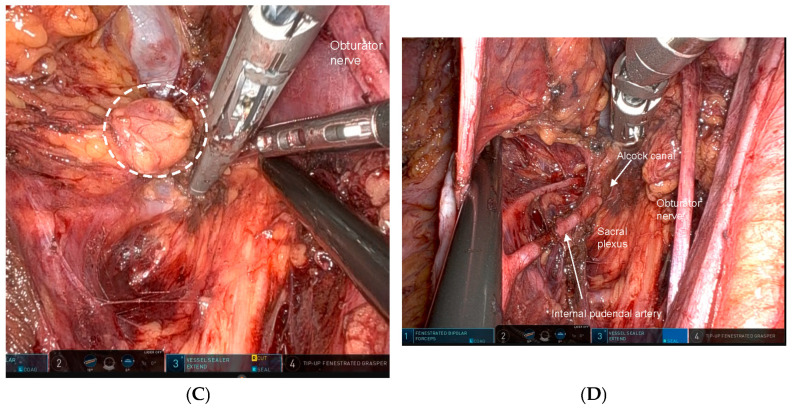
Representative case of metastasis to the distal internal iliac lymph node (station 263D), the most common site of lateral pelvic involvement in advanced lower rectal cancer. (**A**) Pelvic CT demonstrates a clearly enlarged lymph node along the Alcock canal (arrow). (**B**) Pelvic MRI confirms a suspicious node located inferior to the superior vesical artery and adjacent to the inferior vesical vessels (arrow). (**C**) Intraoperative robotic view of the left lateral pelvic compartment showing a prominently swollen station 263D node (dotted circle). (**D**) Post-dissection view following complete excision of the station 263D lymphatic basin, including removal of the enlarged node, which was subsequently confirmed as metastatic on pathological examination.

The caudal and dorsal extents of dissection are confirmed by identifying key anatomic landmarks, including the coccygeus muscle, the tendinous arch of the levator ani, the levator ani muscle itself, and the internal pudendal artery as it enters the Alcock canal. The distal margin of the 263D field is then connected seamlessly with the caudal aspect of the obturator compartment (station 283), allowing residual nodal tissue to be mobilized from the inferior vesical vessels and ensuring complete clearance of the distal internal iliac basin. Figure 3 illustrates a representative case of metastasis to the distal internal iliac lymph node (station 263D), the most commonly involved lateral pelvic site in advanced lower rectal cancer.

## 5. Conclusions

Robotic technology has fundamentally transformed the feasibility, precision, and global acceptance of LPLND for advanced rectal cancer. While appropriate indication remains the cornerstone of lateral pelvic management, the ability to accurately execute LPLND—particularly in anatomically complex and nerve-dense pelvic spaces—depends heavily on surgical technique. By providing stable high-definition magnification and articulating instruments, the robotic platform allows surgeons to identify multilayered pelvic fascial planes with clarity and to preserve autonomic nerves with a level of precision that is difficult to achieve using conventional laparoscopy or open surgery. These technical advantages facilitate the delicate balance between oncologic radicality and functional preservation that lies at the core of optimal rectal cancer surgery.

As contemporary evidence—including large multicenter analyses from the Lateral Node Study Consortium—continues to validate the oncologic benefit of selective LPLND, Western institutions increasingly recognize its value, particularly for patients with persistent lateral nodes after neoadjuvant chemoradiotherapy. Robotic systems have lowered technical barriers and improved reproducibility, thereby accelerating global adoption and narrowing long-standing East–West differences in treatment strategy. Robotic LPLND offers a unified pathway toward global consensus in rectal cancer care—where precision, preservation, and oncologic rigor coexist. Nevertheless, it should be acknowledged that most available evidence on robotic LPLND remains retrospective, underscoring the need for prospective and multinational validation.

## Figures and Tables

**Table 1:** The current recommendations on LPLND across the JSCCR (2024), NCCN (2025), and ESMO (2025) guidelines.

Feature	JSCCR (Japan) 2024	NCCN (US) 2025	ESMO (Europe) 2025
LLN Classification	Regional lymph node metastasis (N3).	Often considered distant metastasis or systemic spread.	Traditionally systemic, but now partially regionalized in selective contexts.
Primary Strategy	Upfront TME ± LPLND; surgical clearance central to local control.	Neoadjuvant therapy + TME.	Neoadjuvant therapy + TME, with selective LLND.
LPLND Status	Routinely recommended for defined high-risk tumors.	Not routinely recommended.	Selectively recommended for residual disease after nCRT/TNT.
Specific Indications	For tumors distal to peritoneal reflection and cT3–4	Only if nodes are clinically suspected on imaging.	LPLN with a short-axis diameter ≥7 mm before neoadjuvant therapy
Evidence Basis	JCOG0212	Reliant on CRT efficacy; concern for morbidity of LPLND.	Lateral Node Study Consortium

ESMO: European Society for Medical Oncology, JSCCR: Japanese Society for Cancer of the Colon and Rectum, LPLN: lateral pelvic lymph node, LPLND: lateral lymph node dissection, NCCN: National Comprehensive Cancer Network, nCRT: neoadjuvant chemoradiotherapy, TME: total mesorectal excision.

## Abbreviations

ESMO	European Society for Medical Oncology
JSCCR	Japanese Society for Cancer of the Colon and Rectum
LPLN	lateral pelvic lymph node
LPLND	lateral lymph node dissection
ME	mesorectal excision
NCCN	National Comprehensive Cancer Network
nCRT	neoadjuvant chemoradiotherapy
TME	total mesorectal excision
TNT	total neoadjuvant therapy
